# SPARC-induced increase in glioma matrix and decrease in vascularity are associated with reduced VEGF expression and secretion

**DOI:** 10.1002/ijc.23450

**Published:** 2008-03-18

**Authors:** Christopher K Yunker, William Golembieski, Nancy Lemke, Chad R Schultz, Simona Cazacu, Chaya Brodie, Sandra A Rempel

**Affiliations:** 1 Barbara Jane Levy Laboratory of Molecular Neuro-Oncology, Hermelin Brain Tumor Center, Department of Neurosurgery, Henry Ford HospitalDetroit, MI; 2 William and Karen Davidson Laboratory of Cell Signaling and Tumorigenesis, Hermelin Brain Tumor Center, Department of Neurosurgery, Henry Ford HospitalDetroit, MI

**Keywords:** SPARC, matrix, collagen I, vascularity, VEGF(R1/2), gliomas

## Abstract

Glioblastomas are heterogeneous tumors displaying regions of necrosis, proliferation, angiogenesis, apoptosis and invasion. SPARC, a matricellular protein that negatively regulates angiogenesis and cell proliferation, but enhances cell deadhesion from matrix, is upregulated in gliomas (Grades II–IV). We previously demonstrated that SPARC promotes invasion while concomitantly decreasing tumor growth, in part by decreasing proliferation of the tumor cells. In other cancer types, SPARC has been shown to influence tumor growth by altering matrix production, and by decreasing angiogenesis *via* interfering with the VEGF-VEGFR1 signaling pathway. We therefore examined whether the SPARC-induced decrease in glioma tumor growth was also, in part, due to alterations in matrix and/or decreased vascularity, and assessed SPARC-VEGF interactions. The data demonstrate that SPARC upregulates glioma matrix, collagen I is a constituent of the matrix and SPARC promotes collagen fibrillogenesis. Furthermore, SPARC suppressed glioma vascularity, and this was accompanied by decreased VEGF expression and secretion, which was, in part, due to reduced VEGF165 transcript abundance. These data indicate that SPARC modulates glioma growth by altering the tumor microenvironment and by suppressing tumor vascularity through suppression of VEGF expression and secretion. These experiments implicate a novel mechanism, whereby SPARC regulates VEGF function by limiting the available growth factor. Because SPARC is considered to be a therapeutic target for gliomas, a further understanding of its complex signaling mechanisms is important, as targeting SPARC to decrease invasion could undesirably lead to the growth of more vascular and proliferative tumors. © 2008 Wiley-Liss, Inc.

Glioblastomas are the most malignant grade of glioma.^1^ Treatment of these tumors is difficult, because of their high degree of heterogeneity. Heterogeneity is present at the cellular level, where different clonal populations may have arisen over time, and this makes it difficult to design a single treatment that will attack all tumor cells.^1^, ^2^ Heterogeneity also occurs at the phenotypic level where, simultaneously, different regions within the tumor may be undergoing different biological processes such as necrosis, tumor cell migration, proliferation, angiogenesis and apoptosis and invasion into the adjacent brain.^1^

Present-day capabilities of interrogating the genetic and protein profiles of tumors and identifying entire altered signaling pathways make it possible to better design therapeutic approaches to target key proteins or their downstream effectors. Such studies indicate that the cancer profile is closely related to the embryonic profile,^3^ suggesting that many of the cancer-related genes are developmental genes that have been inappropriately re-expressed. For gliomas, one such protein is secreted protein acidic and rich in cysteine (SPARC), also known as osteonectin or BM-40.

SPARC is a secreted glycoprotein belonging to the matricellular family of proteins that mediate cell-matrix interactions that affect diverse biological functions including proliferation, survival, adhesion and migration.^4^ They are expressed during embryonic development, and in adult tissues their expression is limited to tissues undergoing repair or remodeling due to wound healing or natural processes.^5^ In the developing brain, SPARC is expressed in angiogenic microvasculature,^6^ and in the adult brain it is expressed in the locus coeruleus,^7^ ganglion cells, and astrocytes of the adult retina,^8^ but not in cells of the cerebral cortex.^9^

We have previously confirmed that SPARC protein is undetectable in normal adult cerebral cortex; however, it was found to be highly expressed in gliomas of all grades, in both the tumor cells and angiogenic endothelial cells, and it was upregulated in angiogenic endothelial cells and reactive astrocytes in the tumor-adjacent brain tissue.^10^ We and others have demonstrated that SPARC promotes invasion *in vitro*^11^, ^12^ and *in vivo*.^13^, ^14^ We demonstrated that SPARC-induced tumor invasion was associated with decreased tumor cell proliferation and overall tumor volume,^13^ suggesting that the ability of SPARC to negatively impact cell growth may be by promoting a migratory *versus* a proliferative phenotype^15^ in gliomas.

The negative effects of SPARC on tumor growth that result from its inhibition of tumor cell proliferation are likely complemented by the ability of SPARC to negatively affect endothelial cell proliferation.^16^, ^17^ This modulation may be accomplished in part by inhibiting growth factor signaling pathways, including those regulated by VEGF,^5^ which is a major contributor to glioma angiogenesis.^18^ SPARC has been shown to negatively regulate endothelial cell proliferation by attenuating VEGF-VEGFR1 signaling by binding to VEGF and inhibiting the growth factor binding to its receptor.^19^ Recent data, however, indicate that VEGF-VEGFR signaling is not restricted to endothelial cells, as the receptors for VEGF have been identified on tumor cells,^20^ including human glioma tissues,^20^, ^21^ primary glioma cells^20^ and established cell lines.^21^ This suggests that SPARC could negatively impact not only glioma angiogenesis but also glioma cell proliferation and overall tumor growth through inhibition of the VEGF-VEGFR signaling pathway.

SPARC also affects matrix composition. Depending on the matrix concentration and regional expression within a tumor,^22^ the matrix may affect cytokine regulation of endothelial cell proliferation.^23^ For example, SPARC promotes the synthesis and secretion of several collagens including collagen I.^24^ When GBM spheroids were grown in collagen matrices, increasing collagen I concentration correlated with decreased spheroid growth *in vitro*.^22^ *In vivo*, collagen I decreased glioma tumor growth in a flank model.^25^ In relevance to this study, Chlenski *et al*. found that SPARC impaired tumor growth of human embryonic kidney 293 xenograft tumors, and this was accompanied by inhibited angiogenesis and increased collagen I.^26^

These studies suggest that the multiple effects of SPARC on endothelial and tumor cell growth and changes to the tumor microenvironment likely combine to thwart angiogenesis and tumor growth. Therefore, we hypothesized that the previously reported suppressed tumor growth in the SPARC-transfected glial tumors,^13^ which was accompanied by decreased tumor proliferation,^13^ might be accompanied by increased matrix production and inhibition of tumor vascularity. Furthermore, we hypothesized that SPARC inhibition of the VEGF-VEGFR signaling in endothelial and/or glioma cells would be involved. To test this hypothesis, control- and SPARC-transfected glioma cells were assessed *in vivo* and *in vitro* for SPARC-induced changes in matrix production, vascularity, VEGF-VEGFR expression and SPARC-VEGF interaction.

## Material and methods

### Cell culture and reagents

Standard tissue culture reagents were purchased from Gibco-BRL (Gaithersburg, MD). Fetal bovine serum (FBS), Superscript First-Strand Synthesis System, Platinum Taq DNA Polymerase, SeeBlue Plus 2 and MagicMark XP Western Standards were obtained from Invitrogen (Carlsbad, CA). Noble agar was purchased from Difco Laboratories (Livonia, MI). The BCA protein assay kit was purchased from Pierce Chemical (Rockford, IL). Anti-SPARC (Haematologic Technologies, Essex Junction, VT, #AON5031), anti-human procollagen I (Chemicon, Millipore, Bedford, MA, #MAB1912), anti-factor VIII (Dako, Carpinteria, CA, #A0082), anti-VEGF (Santa Cruz, Santa Cruz, CA, #SC-7269), anti-VEGF (clone 12D7, a kind gift from Dr. Rolf Brekken), VEGFR1/Flt-1 (Santa Cruz, CA, #SC-316), VEGFR2/FLK-1 (Santa Cruz, CA, #SC-6251), VEGFR2/FLK-1 (LabVision/Neomarkers, Freemont, CA, #RB-9239) and anti-actin (Santa Cruz, CA, #SC-1616) were obtained as indicated. ECL kits were purchased from Amersham Biosciences (Piscataway, NJ). Immobilon P membranes were purchased from Millipore (Bedford, MA) and blotting grade blocker non-fat dry milk was from Bio-Rad Laboratories (Hercules, CA). RNeasy Lipid Tissue mini kit was purchased from Qiagen (Valencia, CA) and protein G-agarose was from Roche Diagnostics (Indianapolis, IN).

### Control and SPARC-transfected U87MG cells

Derivation of the U87MG-derived parental control clone P [U87T2], the P-derived SPARC-transfected clones (S1 [C2A4] and S2 [A2b2]) and P-derived vector controls (VC1 and VC2) is as previously reported.^11^ The SPARC- and vector-transfected clones were maintained in 400 μg/ml neomycin (G418) and 1 μg/ml puromycin, whereas the parental control clone was maintained only in G418.

### Rat brain xenograft implantation and tissue processing

Vector control- and SPARC-transfected cells (5 × 10^5^) were implanted using IACUC-approved protocols as previously reported.^13^ Rats were sacrificed 7 days later. The rats were anesthetized, and death followed *via* cardiac puncture (*i.e*., perfusion with 250–400 ml of sterile 0.9% saline solution and fixation with 250–400 ml of 10% formalin). The brains were removed and stored in 10% formalin for at least 24 hr. Formalin-fixed rat brains were placed in a 200–400 g coronal rat brain matrix (Activational Systems, Warren, MI) and sliced into 2-mm blocks. These blocks were then routinely processed, paraffin-embedded and serially sectioned at 5 μm. *n* = 3–6 rats/clone.

### Diastase digestion and periodic acid Schiff reaction

Periodic acid Schiff (PAS) staining was performed as published^25^ with minor modifications. The 5-μm sections were dried in a 60°C oven for 1 hr and routinely deparaffinized to water. Untreated sections (water) and paired sections in diastase solution (0.1 g/100 ml water) were incubated for 1 hr at 37°C. Sections were washed in running water for 5 min and placed in 0.5% periodic acid solution. Sections were then rinsed in four changes of distilled water, incubated in Schiff's reagent for 15 min and washed well in water for 10 min. Sections were then stained in Biocare hematoxylin for 15 s, washed in water, placed in ammonia water to blue, washed, dehydrated through 95% (2 times) and 100% ethanol (2 times), cleared in xylene (2 times) and mounted. Liver sections (± diastase) were used as controls.

### Picrosirius red staining

Staining was performed as published^26^–^28^ with minor modifications. Sections were deparaffinized and hydrated to water and exposed to 0.1% picrosirius red for 1 hr at room temperature, and washed in 1% acidified water twice for 15 min each. Sections were washed, dehydrated in alcohols, cleared in xylene and mounted. Sections are examined with polarized light microscopy. Thicker and more closely packed fibrils are orange to red, whereas thinner fibrils are yellow to green.^28^

### Immunohistochemistry

Immunohistochemistry was performed using the Biocare Medical Nemesis 7200 (Concord, CA) stainer and reagents for anti-SPARC antibody (1:13,333 dilution in 0.25% BSA in PBS), and anti-procollagen I antibody (1:250 dilution), anti-factor VIII (1:1,200), anti-VEGF antibody (#SC-7269, 1:250) or anti-VEGFR2 antibody (1:3,000) were all diluted in DaVinci antibody diluent. The 5-μm sections were dried in a 60°C oven for 1 hr and routinely deparaffinized to water. SPARC and VEGFR2 were detected after heat-induced antigen retrieval using citrate buffer (pH 6.0) as previously described.^10^, ^13^ VEGF, factor VIII and procollagen I were detected after treatment with 0.4% pepsin in 0.01 N HCl at 37°C for 50, 90 or 45 min, respectively. Control sections were processed omitting the primary antibody and substituting with the appropriate immunoglobulin isotype.

### Endothelial cell counts

For each tumor, sections were scanned at 10× magnification to identify hotspots or areas of densest factor VIII staining. Then 2–6 fields/section (depending on tumor area) were captured at 40× magnification. To count factor VIII-positive cells, images at 40× magnification were printed as 8.5 by 11″ photographs. Any single cell that stained positively for factor VIII in a field was counted in each case. A visible lumen was not required.^29^ All images were counted by 3 investigators, and the values were averaged. For each cell line, 3–6 tumors were assessed and averaged. The results are presented as the average number of factor VIII-positive endothelial cells/40× magnification/clone ± the SD.

### Media and lysate preparation

For monolayer culture, equal numbers of cells were plated for 72 hr before media and lysates were collected. For spheroid culture, cells (4 × 10^6^) were seeded on agar-coated T75 flasks for 72 hr as previously reported.^11^ Conditioned media were collected in serum-free conditions with constant medium volumes, and protein lysates were extracted with a single-detergent lysis buffer as previously reported.^30^, ^31^ The protein concentration was determined using the BCA protein assay. Media and lysates were used for the coimmunoprecipitation and Western blot analyses.

### Coimmunoprecipitation of VEGF and SPARC

One milliliter of lysate containing 200–500 μg of total protein was precleared overnight with 25 μl of protein G-agarose beads. Either 2.7 μg of VEGF antibody (#PC315), 5.0 μg VEGF antibody (clone 12D7^32^) or 1 μg of mouse monoclonal SPARC antibody (#AON5031) was added to the precleared lysates, and the samples were then incubated at 4°C with mixing for 1 hr. Protein G-agarose beads (25 μl) were added and the samples were incubated with mixing overnight at 4°C. The next day, the beads were microcentrifuged for 20 s at 12,000*g*, and washed with 1 ml of 1× lysis buffer for 20 min at 4°C with mixing. This was repeated for a total of 3 washes. After removing the final wash, 30 μl of 2× SDS gel loading buffer was added, and the proteins were denatured by heating to 100°C for 3 min. The protein G-agarose was removed by centrifugation for 20 s at 12,000*g* at room temperature, and the supernatants were transferred to fresh tubes. Immunoprecipitated proteins were subjected to 13.5% SDS-polyacrylamide gel electrophoresis and processed as described under Western blot analysis.

### Immunoprecipitation of VEGFR2

Spheroid lysates (1 ml of 300 μg/ml) were precleared as described previously and then incubated with either 5 μg of anti-Flt-1 (#SC316) or anti-Flk-1 (SC6251) antibodies for 1 hr. Protein-G agarose beads (25 μl) were then added to each tube and incubated and washed as described earlier. SDS gel loading buffer (35 μl) was added and the samples were boiled for 3 min at 100°C and centrifuged to remove beads. Samples were electrophoresed through 7.5% SDS-polyacrylamide gels, transferred, and subjected to Western blot analysis as follows.

### Western blot analysis

For total proteins, media (21 μl) and lysates (18 μg) were subjected to 13.5% SDS-polyacrylamide gel electrophoresis. For all Western blots, the molecular weight standard, a mixture of 10 μl MagicMark XP Western Standard and 5 μl SeeBlue Plus 2 prestain standard was run on each gel. Resolved proteins were transferred to an Immobilon P membrane in a Tris-glycine transfer buffer (48 mM Tris, 39 mM glycine, 20% v/v methanol; pH 9.2).

Membranes for total and immunoprecipitated proteins were dried at room temperature, re-wet in methanol, rinsed in Tris-buffered saline ([TBS]; pH 7.5) and then blocked for 1 hr at room temperature in 5% blotting grade non-fat dry milk blocker in TBS. The membranes were incubated with anti-SPARC (1:8,000; #AON5031), anti-VEGF (1:500; #SC-7269), anti-VEGFR1 (1;100; #SC316) or anti-VGEFR2 (1:200, SC-6251; or 1:500, RB-9239) antibodies in 2.5% blocker in TBS for 1 hr at room temperature. The membranes were then washed twice in TBST (TBS plus 0.1% Tween-20) and twice in 2.5% blocker in TBS. Membranes were incubated with appropriate secondary HRP-linked antibodies (in 2.5% blocker in TBS) for 30 min at room temperature, and then washed 4 times in TBST. SPARC and VEGF proteins were detected using the ECL kit, following the manufacturer's instructions. For loading control, the dried membranes were reprobed for actin (1:500 dilution).

### VEGF and VEGFR RT-PCR

Total RNA was extracted from cell lines and human tissues (collected under an approved IRB protocol) using the RNeasy Lipid Tissue mini kit, according to the manufacturer's protocol and as previously reported.^33^ cDNA synthesis was performed using Superscript First-Strand Synthesis System with oligo(dT) at 42°C for 50 min. PCR was performed with Platinum Taq DNA Polymerase and gene-specific primers (Table I) for VEGF,^34^ VEGF165,^35^ VEGFR1^36^ and VEGFR2^36^ using an Eppendorf Master cycler 9600. For VEGF reactions, GAPDH primers^33^ were coamplified as a control for cDNA integrity. An initial denaturation at 94°C for 1 min was followed by 27 cycles of denaturation at 94°C for 30 sec, annealing at 60°C for 1 min, extension at 72°C for 2 min and a single cycle for final extension for 10 min at 72°C. For VEGF165 primers, 94°C for 30 sec was followed by 63°C for 45 sec, 72°C for 1 min for 30 cycles and 72°C for 10 min. For VEGFR1 and VEGFR2 reactions, GAPDH was coamplified with VEGFR1 but amplified separately for VEGFR2 because the product sizes were similar. The initial 94°C for 10 min was followed by 35 cycles of denaturation at 94°C for 30 sec, annealing at 63°C for 45 sec, extension at 72°C for 1 min and a single cycle for final extension for 10 min at 72°C. A negative control included RT-PCR without cDNA synthesis to ensure that no amplification was derived from contaminating DNA. The RT-PCR products were visualized on 1.5% agarose gel with ethidium bromide staining.

**TABLE I:** RT-PCR Primers

		Primer sequence	Ref
RT-PCR			
VEGF	Sense	5′-TCG GGC CTC CGA AAC CAT GA-3′	34
	Antisense	5′-CCT GGT GAG AGA TCT GGT TC-3′)	
VEGF165	Sense	5′-GAG ATG AGC TTC CTA CAG CAC-3′	35
	Antisense	5′-TCA CCG CCT CGG CTT GTC ACA T-3′	
VEGFR1	Sense	5′-CTA GGA TCC GTG ACT TAT TTT TTC TCA ACA AGG-3	36
	Antisense	5′-CTC GAA TTC AGA TCT TCC ATA GTG ATG GGC TC-3′	
VEGFR2	Sense	5′-CCT GGG GTA AAG ATT GAT GAA G-3′	36
	Antisense	5′-AGT TGG GGT GTG GAT GCT-3′	
qRT-PCR			
S12	Sense	5′-TGC TGG AGG TGT AAT GGA G-3′	37
	Antisense	5′-CAA GCA CAC AAA GAT GGG CT-3′	
VEGF165 110 bp	Sense	5′-ATG CGG ATC AAA CCT CAC CAA G-3′	38
	Antisense	5′-GGC CCA CAG GGA TTT TCT TGT CTT GC-3′	
VEGF165 60 bp	Sense	5′-TTC CTA CAG CAC AAC AAA TG-3′	38
	Antisense	5′-CAG GGA TTT CTT GTC TTG C-3′	

### qRT-PCR

Quantitative RT-PCR for VEGF165 was performed as previously reported.^37^ A primer optimization step was tested for each set of primers to determine the optimal primer concentrations. Primers, 10 μl of 2× SYBR Green Master Mix (Invitrogen, Carlsbad, CA) and 8 μl of a 1:30 dilution of the same cDNA used for the RT-PCR analyses were resuspended in a total volume of 20 μl of PCR amplification solution. VEGF165 primers (Table I) were used to amplify 110 bp and VEGF 60 bp fragments. The sense primers are in exon 4 and the antisense primers cover the junction between exons 5 and 6.^38^ S12 was amplified as a control. Reactions were run on an ABI Prism 7000 Sequence Detection System (Applied Biosciences, Foster City, CA). The cycling conditions composed of 4-min polymerase activation at 95°C and 40 cycles, 90°C for 30 sec, 60°C for 30 sec and 72°C for 1 min. Cycle threshold (Ct) values were obtained from the ABI 7000 software. S12 levels were also determined in the same run for each RNA sample as controls. Fold change of relative mRNA expression was determined using the 2^−ΔΔCT^ method as previously reported.^39^ qRT-PCR reaction products were run on an agarose gel to confirm the product size and specificity (data not shown).

### Imaging

Immunohistochemical images (20×, 40× magnification) were captured using a Nikon Eclipse E800M microscope connected to a Nikon DXM1200C digital camera and digitized using ACT-1C software. Polarized images (10× magnification) were captured using Image Pro +5 software using an Evolution MP camera (MediaCybernetics) attached to a Nikon Optiphot 2 microscope. All the composites were assembled using Photoshop.

### Statistical analyses

Sections from 3 to 6 animals per group were used for immunohistochemistry and vascularity calculations. Student's *t*-tests were used to calculate the *p*-values. Significance was set at *p* ≤ 0.05.

## Results

### SPARC increases collagen I production and fibrillogenesis

**FIGURE 1:**
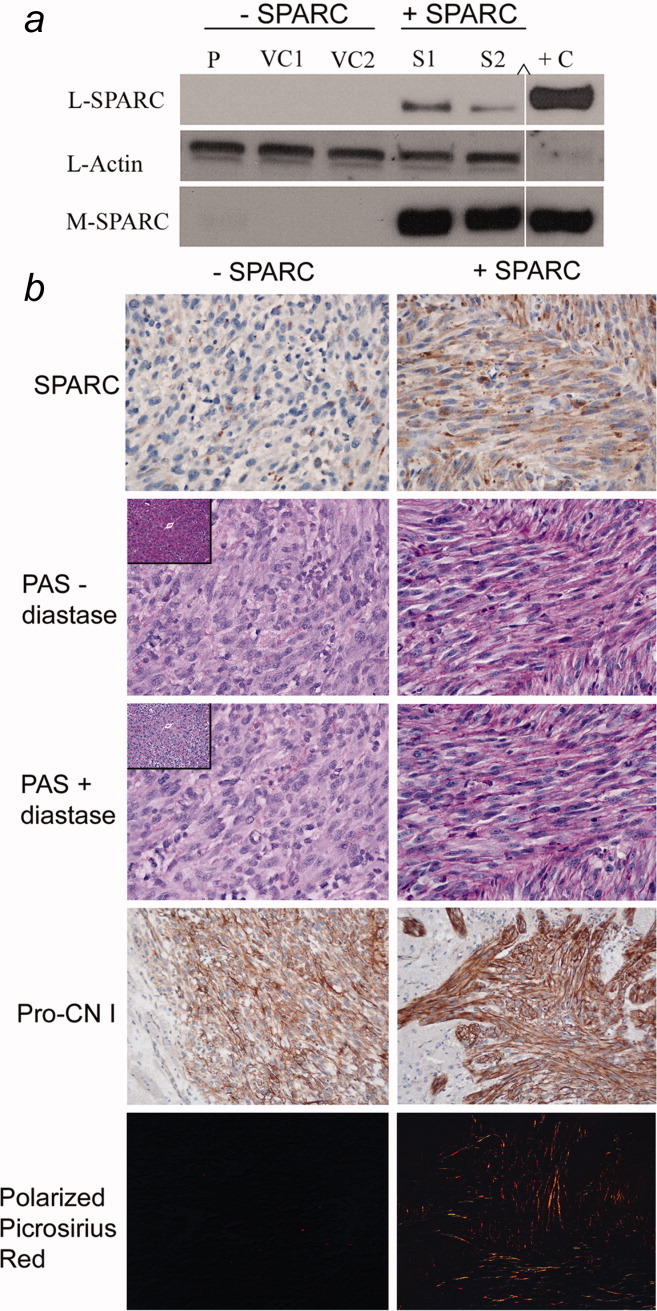
Matrix assessment in control- (−SPARC) and SPARC- (+SPARC) expressing tumors. (*a*) The level of SPARC in the control and SPARC-expressing cells was assessed by Western blot analysis prior to brain implantation. P-parental clonal U87MG-derived cell line, VC1 and VC2-vector control cell lines 1 and 2, S1 and S2-SPARC-transfected cell lines 1 and 2. +C-SPARC protein positive control. L: lysate, M: medium. (∧) indicates that the signal is on the same gel but moved closer. Actin detection on the same lysate blot was used as a loading control. (*b*) Representative images of tumor sections either immunohistochemically stained for SPARC (×40) or procollagen I (Pro-CN I; ×20), or enzyme-treated or untreated sections stained with Schiff's reagent (PAS ± diastase; ×40) or stained with picrosirius red and imaged under polarized light (×20).

SPARC not only increases the synthesis of collagen I,^24^ but also regulates the processing of procollagen I and collagen in dermal fibroblasts.^40^ Therefore, to assess changes in matrix production, processing and fibrillogenesis because of SPARC expression, control- (−SPARC) and SPARC (+SPARC)-expressing U87 glioma cells (Fig. 1*a*) were implanted into nude rat brains, and Day 7 control and SPARC-expressing tumors were obtained (Fig. 1*b*). Tumor sections were subjected to 3 different assays known to detect collagen, including the periodic acid Schiff reaction (PAS) with or without diastase treatment, procollagen I immunohistochemistry and picrosirius red staining with polarized light microscopy (Fig. 1*b*).

PAS stains glycogen and matrix proteins, including collagen, magenta. Diastase treatment removes any glycogen, and therefore comparisons between the staining intensity with or without diastase treatment can be used to characterize tissue constituents. Using this method, we observed no changes in intensity of the PAS staining, comparing sections with *versus* without diastase treatment in either the control tumors or the SPARC-expressing tumors. In contrast, a clear difference was observed for the control liver tissues. These data indicate that glycogen is not a major constituent of the tumors and that the magenta staining is due to matrix proteins. The stronger intensity of PAS staining in the SPARC-expressing tumors compared to the control tumors indicates that the SPARC-expressing tumors have more matrix (Fig. 1*b*).

To determine whether the matrix detected by PAS staining included collagen, tumor sections were subjected to immunohistochemical analysis using an antibody that detects only human procollagen I. Both the control and the SPARC-expressing tumors expressed procollagen I. However, the SPARC-expressing tumors were more intensely stained, suggesting greater levels of procollagen I synthesis (Fig. 1*b*). This was supported by the more intense staining of the SPARC-expressing tumors with picrosirius red, a compound that stains collagen bright pink (data not shown).

Picrosirius red staining is also used to detect immature and mature collagen fibrils under polarized light. When examined under polarized light, only the SPARC-expressing tumors had immature and mature collagen fibers (Fig. 1*b*), suggesting that SPARC expression enhanced collagen I processing and fibrillogenesis.

### SPARC decreases vascularity

**FIGURE 2:**
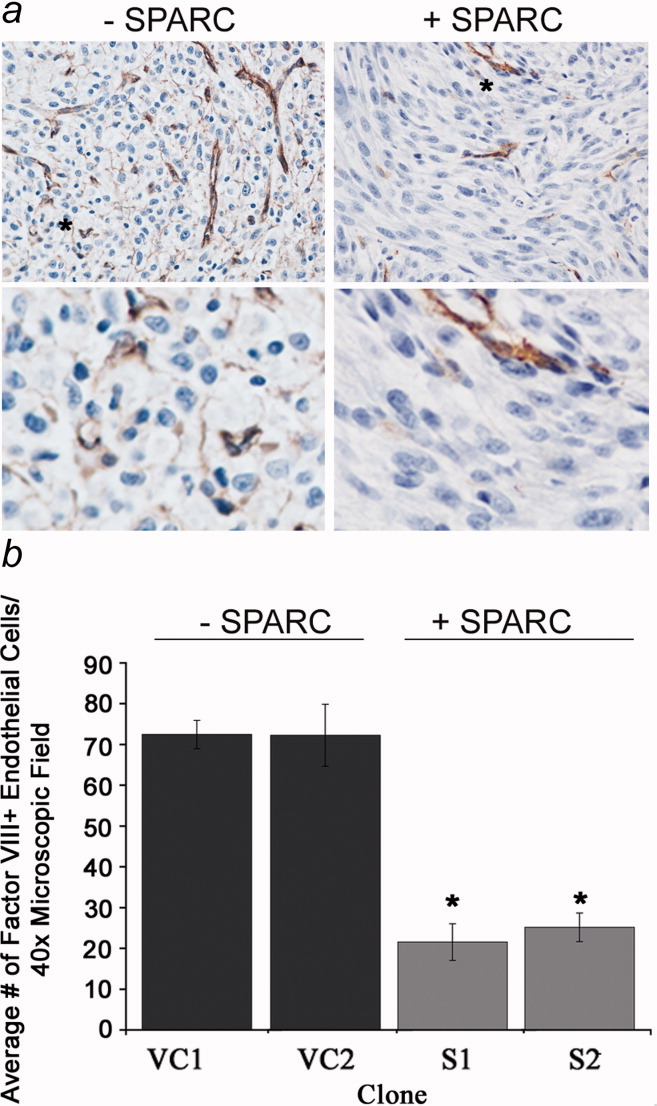
Factor VIII expression in control- (−SPARC) and SPARC- (+SPARC) expressing tumors. (*a*) Representative tumor sections were immunohistochemically stained for factor VIII (top panels: ×40 magnification; bottom panels: ×4 further magnification of the region indicated by an asterisk in the top panels). (*b*) Average number of factor VIII-positive endothelial cells in control *versus* SPARC-expressing tumors. Note that enhanced SPARC expression correlates with significantly reduced factor VIII staining. *p ≤ 0.001.

Because matrix impacts vascularity,^23^ and SPARC-expressing tumors had more matrix, we evaluated the effects of SPARC expression on vascularity. Serial sections were stained with factor VIII (Fig. 2*a*), and the average number of factor VIII-positive endothelial cells/field was measured for each clone and plotted (Fig. 2*b*). The SPARC-expressing tumors had significantly less vascularity than the control tumors (*p ≤ 0.0001).

### SPARC does not mediate paracrine or autocrine signaling through VEGF interaction or VEGF-VEGFR1 signaling

**FIGURE 3:**
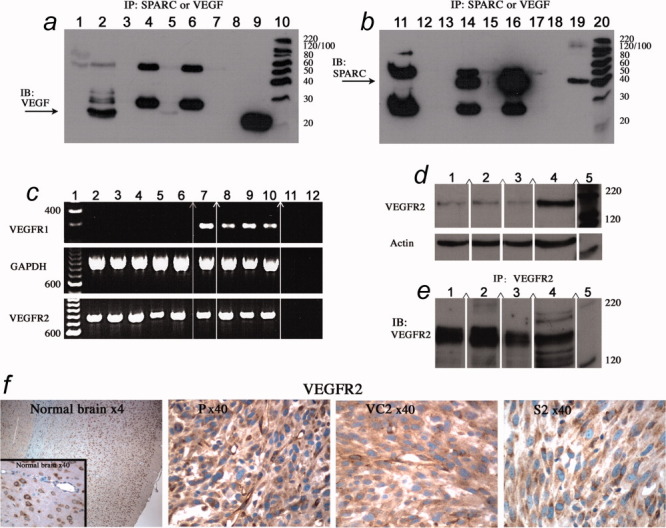
Assessment of SPARC, VEGF and VEGFR expression. (*a* and *b*) Conditioned media (CM) from control (VC2) and SPARC-transfected (S2) spheroids were subjected to immunoprecipitation (IP) with antibody to (Ab) to either VEGF or SPARC, followed by Western immunoblotting (IB) with anti-VEGF antibody (*a*) or anti-SPARC antibody (*b*). (Panel *a*) Lane 1: VEGF antibody alone (control), Lane 2: anti-VEGF IP of VEGF protein (control), Lane 3: IP of VC2-CM minus primary Ab (control), Lane 4: anti-SPARC IP of VC2-CM, Lane 5: anti-VEGF IP of VC2-CM, Lane 6: anti-SPARC IP of S2-CM, Lane 7: anti-VEGF IP of S2-CM, Lane 8: empty lane, Lane 9: VEGF protein (for size standard) and Lane 10: molecular weight standard. Arrow: VEGF antibody immunoprecipitated VEGF only from control VC2-CM. (Panel *b*) Lane 11: SPARC antibody alone (control), Lane 12: empty lane, Lane 13: IP of VC2-CM minus primary Ab (control), Lane 14: Anti-SPARC IP of VC2-CM, Lane 15: Anti-VEGF IP of VC2-CM, Lane 16: Anti-SPARC IP of S2-CM, Lane 17: anti-VEGF IP of S2-CM, Lane 18: empty lane, Lane 19: SPARC protein (for size standard) and Lane 20: molecular weight standard. Arrow: SPARC antibody immunoprecipitated SPARC only from control VC2- and S2-CM. Bands at 55 and 23 KDa are IgG heavy and light chains. Note that no coimmunoprecipitation was observed using either antibody, even when blots were overexposed as illustrated. (*c*) RT-PCR analysis of VEGFR1 (Flt-1) and VEGFR2 (Flk-1). (Top gel) Lane 1: molecular weight standard, Lane 2: control parental U87 cells, Lane 3: vector control VC1, Lane 4: vector control VC2, Lane 5: SPARC-transfected clone S1, Lane 6: SPARC-transfected clone S2, Lane 7: normal brain N141, Lane 8: astrocytoma A203, Lane 9: anaplastic astrocytoma AA152, Lane 10: GBM373, Lane 11: −RT-control, Lane 12: H_2_O Control reaction without cDNA. (Middle gel) GAPDH control coamplification of GAPDH in the same samples used with VEGFR1 primers to confirm integrity of cDNA. Note the lack of VEGFR1 in U87-transfected cells. (Bottom gel) VEGFR2 RT-PCR analysis of the same samples as the top gel. (*d*) Western blot analysis of VEGFR2. Lane 1: SPARC-transfected clone S2, Lane 2: vector-transfected control VC2, Lane 3: parental U87, Lane 4: THP-1-positive VEGFR2 control and Lane 5: molecular weight standard. The VEGFR2 blot was stripped and reprobed for actin as control for loading. (*e*) Immunoprecipitation (IP) of VEGFR2 for the same samples in panel *b*, followed by Western blotting (IB) for VEGFR2. VEGFR2 is present in U87 parental and transfected cells. (*c*–*e*) (∧) Indicates that the signal is on the same gel but moved closer. (*f*) VEGFR2 immunohistochemistry in normal rat brain,^41^ controls (P, VC2) and SPARC-transfected S2 tumor. Magnifications as indicated. VEGFR2 transcript and protein are present in the U87 control and SPARC-transfectants.

SPARC is capable of binding to VEGF, and in doing so, specifically inhibits VEGF-VERGFR1 signaling of endothelial cells.^17^ Gliomas also express VEGFR1.^20^, ^21^ Therefore, we evaluated the status of SPARC-VEGF interaction by coimmunoprecipitation and the status of VEGFR1 expression in our glioma model. We hypothesized that SPARC would bind to VEGF and inhibit VEGF-VEGFR1-induced angiogenesis and tumor growth, as we observed a decrease in tumor growth^13^ and vascularity. We found no coimmunoprecipitation of the 2 proteins in either lysates (data not shown) or media of control or SPARC-transfected cells (Figs. 3*a* and 3*b*), although each antibody was capable of immunoprecipitating its cognate protein. These results were then confirmed using another VEGF antibody^32^ previously reported to coimmunoprecipitate VEGF and SPARC (data not shown). In addition, experiments using 4-times concentrated media were also negative for coimmunoprecipitation (data not shown). We also demonstrated that the glioma cell lines used do not express VEGFR1 (Fig. 3*c*). The lack of coimmunoprecipitation with VEGF indicates that the decrease in vascularity induced by SPARC is not because of the inhibition of VEGF-VEGFR paracrine signaling in endothelial cells. Furthermore, the lack of coimmunoprecipitation with VEGF and the lack of VEGFR1 on the glioma cells indicate that the decrease in vascularity induced by SPARC is also not because of the inhibition of VEGF-VEGFR1 autocrine signaling in glioma cells.

### The U87 cell lines express VEGFR2

In a proposed mechanism involving SPARC-integrin interaction, SPARC upregulates VEGF expression and autocrine stimulation of prostate cancer cell growth *via* VEGF-VEGFR2 signaling.^42^ Such a mechanism could lead to a large increase in VEGF expression that could also effect overall vascularity by disrupting vessel integrity. Therefore, we examined our cells for VEGFR2 expression. RT-PCR analysis demonstrated the presence of VEGFR2 transcripts in the U87-derived cell lines (Fig. 3*c*). This was confirmed by Western blot analysis (Fig. 3*d*) and immunoprecipitation (Fig. 3*e*). Finally, immunohistochemistry (Fig. 3*f*)^41^ demonstrated that VEGFR2 expression was observed in tumor cells *in vivo*.

### SPARC does not mediate paracrine VEGF-VEGFR2 signaling by increased secretion of VEGF

**FIGURE 4:**
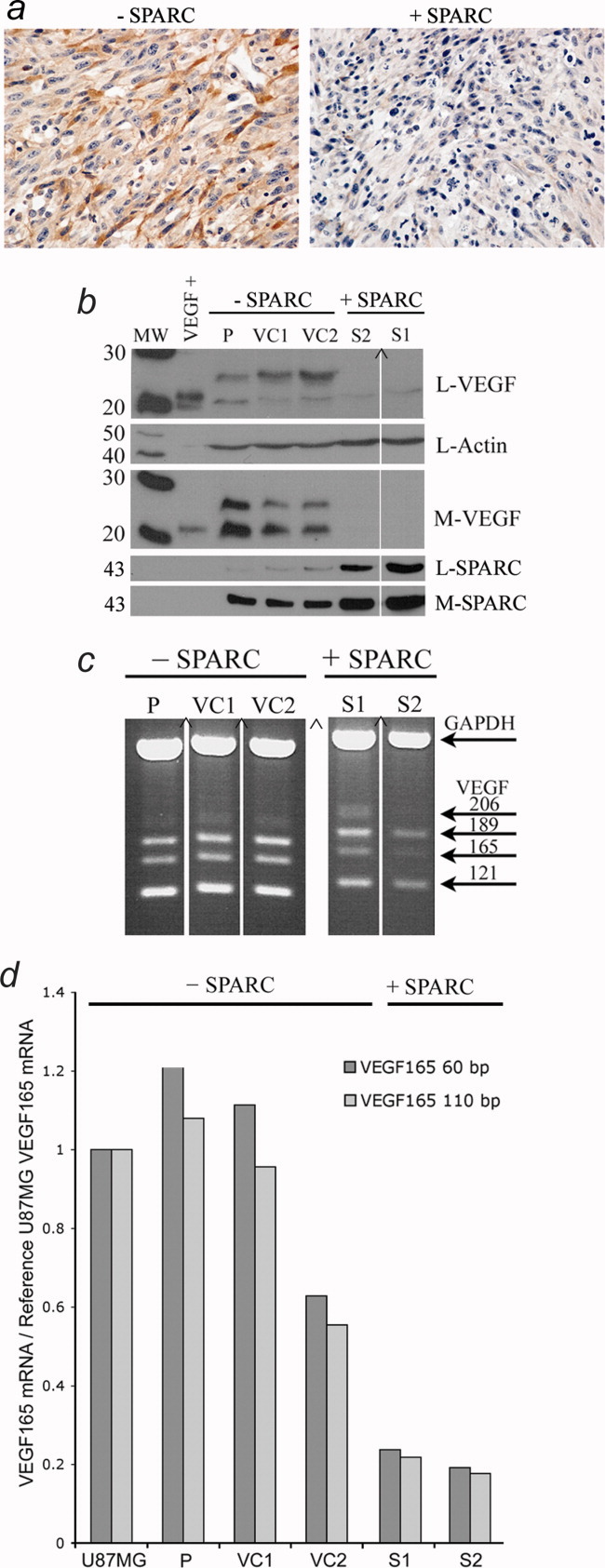
Immunohistochemical, Western blot and RT-PCR analysis of VEGF protein and transcripts in control- (−SPARC) and SPARC- (+SPARC) transfected clones. (*a*) Representative tumor sections immunohistochemically stained for VEGF expression (×40). Note the decreased VEGF expression in the SPARC-expressing tumors. (*b*) Control and SPARC-expressing spheroids were assessed for VEGF and SPARC expression and secretion by Western blot analysis. L: lysate, M: medium. The same blot was used for all lysate analyses. Actin detection was used as a loading control. Note that increased SPARC expression correlated with decreased VEGF expression and secretion. (*c*) RT-PCR was performed to detect all 4 major VEGF isoforms as indicated. (*b* and *c*) (∧) Indicates that the signal is on the same gel but moved closer. (*d*) Real-time RT-PCR analysis of the VEGF165 isoform. Note that enhanced SPARC expression is associated with decreased VEGF165 transcript abundance. P-parental clonal U87MG-derived cell line, VC1- and VC1-vector control cell lines, S1- and S2- SPARC-transfected cell lines.

Because the cell lines expressed VEGFR2, and SPARC has been implicated in upregulating VEGF expression,^42^ we determined whether increased SPARC expression correlated with increased VEGF expression. Low endogenous SPARC expression was confirmed in the tumor cells of control tumors, and increased expression of SPARC was observed in the SPARC-transfected tumor cells (Fig. 1*b*). Conversely, high levels of VEGF were observed in the controls and much lower levels were observed in the SPARC-transfected tumors (Fig. 4*a*). The data suggest that SPARC decreases tumor growth by suppressing VEGF expression and secretion, thereby attenuating the VEGF-VEGFR1/2 signaling mechanisms.

### Enhanced SPARC expression correlates with decreased VEGF expression and secretion in vitro

To determine whether the decrease in VEGF observed *in vivo* was due to direct SPARC expression and not an environmental influence, the cells were assessed *in vitro* using spheroid culture. VEGF expression and secretion was observed in the control cells (P, VC1, VC2); however, less protein was observed in the lysates (L-VEGF) of SPARC-transfected cells and none was secreted (M-VEGF) in the SPARC-transfected cells (Fig. 4*b*). Two VEGF isoforms were expressed in the control cells, and in comparison with the positive control (VEGF165), are presumed to be VEGF165 and VEGF189.

### SPARC expression correlates with a decrease in VEGF165 transcript

RT-PCR was performed to determine whether the decrease in VEGF protein correlated with the decreased transcript. All 4 major isoforms (206, 189, 165 and 121) were detected in the control and SPARC-transfected cells (Fig. 4*c*). However, increased SPARC expression correlated with a specific reduction in the VEGF165 transcript, which was confirmed using real-time RT-PCR and 2 sets of VEGF165-specific primers (Fig. 4*d*).

## Discussion

In our study, we made the following observations. *In vivo*, using xenograft implantation of U87MG control- and SPARC-transfected clones, we found that increased SPARC expression correlated with increased matrix production, procollagen I expression and collagen I fibrillogenesis. Increased matrix was associated with decreased microvascularity, which corresponded to decreased VEGF expression. *In vitro*, we found that SPARC reduced VEGF expression and secretion, and that this was, in part, accomplished by reduced VEGF165 transcript abundance.

We have previously demonstrated that enhanced SPARC expression increases glioma invasion and concomitantly decreases glioma growth and tumor volume.^13^ The decrease in growth was, in part, due to a decrease in tumor cell proliferation. However, as SPARC is a negative regulator of angiogenesis, we proposed that SPARC could affect overall glioma growth by affecting vascularity. As SPARC also affects matrix synthesis, and matrix can have a profound affect on vascularity,^23^ we determined the effects of SPARC on glioma matrix production.

We found that the number of factor VIII-positive endothelial cells was significantly lower in the SPARC-expressing tumors indicating that SPARC reduced tumor vascularity. In addition, the SPARC-expressing tumors were found to produce more matrix as assessed by PAS staining. This increase was, in part, due to an increase in procollagen I synthesis as determined by immunohistochemistry, and increased collagen I deposition and fibrillogenesis as determined by polarized light microscopy of picrosirius red-stained sections.

Our results agree with those from the Cohn Laboratory.^26^ Their data indicated that SPARC enhanced matrix production and decreased angiogenesis of human embryonic kidney 293 cells implanted into the flanks of athymic nude mice. However, our results do differ with respect to the source of collagen I, which was shown to be from host stromal cells in their model and from tumor cells in our model. Since U87 cells are known to express collagen I and as we used an antibody that detects only human procollagen I, we conclude that the source of collagen I in our tumor model was from the human tumor cells. Differences may be due to tumor type or microenvironmental influences as we used orthotopic implantation *versus* flank implantation.

Collagen I has been associated with decreased T98G glioma growth and invasion *in vivo*.^25^ However, those *in vivo* studies were subcutaneous flank implantations and the microenvironment is vastly different from the brain, which could account for a lack of invasion. Interestingly, it has been shown that collagen concentration and regional expression in the tumor are important in its function.^25^ Using GBM spheroids, high collagen concentration correlated with decreased growth in the spheroid core and increased migration of tumor cells at the periphery. These observations correlate well with our model, in which enhanced SPARC expression decreases overall tumor growth and increases invasion.^13^

SPARC has been implicated in 2 mechanisms of VEGF-VEGFR signaling, which can affect both autocrine and paracrine signaling. In 1 mechanism, SPARC binds directly to VEGF, which inhibits VEGF binding to VEGFR1,^19^ a receptor present on endothelial and some tumor cells. In characterizing our tumor model, we found that the U87-derived glioma cells expressed and secreted VEGF. However, these cells do not express VEGFR1, an observation in agreement with another report.^20^ This lack of receptor expression, therefore, precludes a VEGF-VEGFR1 mechanism that could be disrupted by SPARC in the tumor cells. Exploring the possibility that SPARC binding to VEGF could decrease vascularity by attenuating VEGF-VEGFR1 signaling in endothelial cells, we investigated whether SPARC and VEGF coimmunoprecipitated. We saw no interaction between the proteins, eliminating this mechanism as well. The lack of coimmunoprecipitation is not surprising in light of the inverse correlation of SPARC and VEGF expression, as discussed later.

In the second proposed mechanism, SPARC expression in prostate tumor cells induces an increase in VEGF expression, which upregulates VEGF binding to VEGFR2 present on the tumor cells, thereby promoting tumor growth.^42^ However, the authors found the opposite results using M21 melanoma cells and suggested that these differences are because of the cell type-specific differences. In this study, the U87-derived cells demonstrated the presence of VEGFR2, a result that contrasts another report,^20^ perhaps, because of the differences in the efficacy of the primers used. Therefore, we further validated our RT-PCR observations using Western blot, immunoprecipitation and immunohistochemical analyses. The presence of VEGFR2 and the secretion of VEGF by the control tumors may indeed contribute to their growth. However, the effect of SPARC on these cells was to decrease VEGF expression, an observation that agrees with that reported for the melanoma cells.^42^ Therefore, we conclude that in gliomas, SPARC decreases VEGF secretion and thereby attenuates the VEGFR-VEGFR2 signaling in the U87 glioma cells, which corresponds with the decreased growth previously observed.^13^

We investigated the mechanism whereby SPARC decreases VEGF expression, and found that SPARC suppresses VEGF165 at the transcript level and VEGF protein expression and secretion. The exact mechanisms involved are not known, but SPARC is known to alter the expression of other genes.^33^ In addition, several reports show an inverse relationship between SPARC and VEGF expression; the more SPARC, the less VEGF, as well as the more VEGF, the less SPARC.^43^–^46^ Indeed, a study examining progression of serially passaged primary human glioma cells demonstrated that early passage tumor formation was accompanied by invasion, SPARC expression and no VEGF expression. In contrast, later passage tumors were not invasive, did not express SPARC, but had high levels of VEGF expression.^47^

In summary, these data demonstrate that SPARC upregulates glioma ECM, collagen I is a constituent of this matrix and SPARC facilitates collagen fibrillogenesis. This increase in collagen I correlates with the suppressed tumor growth and increased invasion previously reported.^11^ In addition, SPARC suppression of glioma vascularity is accompanied by decreased VEGF expression and secretion. These data suggest another mechanism of action, whereby SPARC regulates VEGF expression rather than its function. A further understanding of the mechanisms involved is important for utilizing these proteins as therapeutic targets, as inhibiting SPARC alone could undesirably lead to more angiogenic and proliferative tumors and, conversely, targeting VEGF alone might facilitate more invasive tumors.

## Abbreviations

ECM: extracellular matrix
GBM: glioblastoma
PAS: periodic acid Schiff
qRT-PCR: quantitative reverse transcription-polymerase chain reaction
SPARC: secreted protein acidic and rich in cysteine
VEGF: vascular endothelial growth factor
VEGFR1/2: vascular endothelial growth factor receptor 1/2
